# High resolution computed tomography in systemic sclerosis: From diagnosis to follow-up

**DOI:** 10.2478/rir-2024-0023

**Published:** 2024-10-21

**Authors:** Roberta Eufrasia Ledda, Corrado Campochiaro

**Affiliations:** Department of Medicine and Surgery, University of Parma, Parma, Italy; Unit of Immunology, Rheumatology, Allergy and Rare Diseases, IRCCS San Raffaele Hospital, Vita-Salute San Raffaele University, Milan, Italy

**Keywords:** interstitial lung disease, systemic sclerosis, high-resolution computed tomography, pulmonary hypertension

## Abstract

Early diagnosis of interstitial lung disease (ILD) and pulmonary hypertension (PH) is crucial in systemic sclerosis (SSc) for both management and treatment. However, diagnosing SSc-ILD can be challenging because symptoms of lung involvement are often non-specific at the early stages of disease. High-resolution computed tomography (HRCT) of the chest is recognized as the most accurate imaging modality for baseline and follow-up evaluation of SSc-ILD. Key features of SSc-ILD on HRCT include a non-specific interstitial pneumonia (NSIP) pattern, with peripheral ground-glass opacities and extensive traction bronchiectasis. Less common HRCT manifestations include usual interstitial pneumonia (UIP) pattern, followed by diffuse alveolar damage (DAD), diffuse alveolar hemorrhage (DAH) and organizing pneumonia (OP). The extent of disease on HRCT is known to relate with prognosis and serial assessments can be helpful in monitoring disease progression or treatment response. We discuss the main chest computed tomography (CT) manifestations of SSc, highlighting the role of imaging at both baseline and follow-up evaluations.

## Introduction

Systemic sclerosis (SSc) is a connective tissue disease characterized by vasculopathy, immune system activation and fibrosis of the skin and internal organs.^[1]^ Among the protean manifestations of SSc, lung involvement, in the form of interstitial lung disease (ILD) or in the form of pulmonary hypertension (PH), represents a major clinical concern as both ILD and PH are associated with a reduced quality of life and they have become the leading causes of death for SSc patients.^[2,3]^ This is why international efforts have been made to better understand how to manage these conditions and how to diagnose and monitor patients with SSc who have already been diagnosed or are at risk of developing these two complications.^[2,4]^ While in the past chest X-ray was used to assess ILD in SSc patients, high-resolution computed tomography (HRCT) has progressively replaced the use of conventional radiography both for diagnosis and follow-up.^[5]^ This clearly represents a major clinical advance and a groundbreaking shift in the paradigm on when to use and what kind of information can be obtained by HRCT for SSc patients. Quantification of ILD, radiological progression, differential diagnosis and overall disease monitoring do now rely on HRCT thus making this technique an essential and irreplaceable tool.^[5]^ Moreover, while the role of HRCT for parenchymal lung evaluation is established and well-integrated in the rheumatological community, it can also offer insights into other features associated with SSc such esophageal involvement and is a major clue in the work-up of suspected cases of pulmonary arterial hypertension.^[6]^ This is why rheumatologists should be updated on the most recent clinical applications of HRCT and should develop a critical view of HRCT for the care of SSc patients.

This review aims at discussing the main chest computed tomography (CT) manifestations of SSc, highlighting the role of imaging at both baseline and follow-up evaluations. The relevance of technical acquisition and reconstruction parameters of HRCT imaging is also discussed.

## Lung Involvement in Systemic Sclerosis

SSc is classified according to the 2013 ACR/EULAR criteria^[7]^ and is further subclassified into limited cutaneous (lcSSc; affecting the face, neck, and distal limbs) and diffuse cutaneous SSc (dcSSc; affecting the proximal limbs, abdomen, and chest) forms. Each subset is associated with specific clinical features and autoantibody positivity, providing diagnostic and prognostic insights.^[8]^

The exact prevalence of ILD in SSc patients varies widely and depends on the diagnostic tool used for confirmation.^[9]^ The introduction of HRCT has significantly modified the landscape of SSc-ILD, enabling a greater number of patients to be accurately identified even at the early stages of the disease. Furthermore, the prevalence of SSc-ILD differs significantly according to disease subset, with a higher prevalence in dcSSc (ranging from 30% to 71% of patients) compared to lcSSc (ranging from 21% to 53%).^[10]^ Additionally, specific autoantibodies (anti-topoisomerase I and anti-centromere) either increase or decrease the risk of ILD in SSc^[11]^. However, anti-topoisomerase I positivity does not always correlate with ILD, as up to 50% of anti-topoisomerase I positive patients do not have ILD, nor does it correlate with a more rapid ILD progression.^[11]^

Recent literature has also highlighted how even patients at the later stages of the disease can be affected by ILD, which can be significant even in lcSSc patients.^[12,13]^ Therefore, a tailored approach considering various disease features is necessary for monitoring ILD in SSc patients. This heterogeneity is also evident in SSc-PH, the prevalence of which is highly dependent on disease duration and the diagnostic technique used, particularly when comparing right heart catheterization to echocardiography with right heart catheterization being essential for diagnosing SSc-Pulmonary artery hypertension (PAH).^[14]^ Contrast-enhanced chest CT is though mandatory in the work-up of SSc patients with suspected SSc-PAH to rule out secondary causes of PAH and to correctly discriminate between PAH and ILD-related PH.^[14]^ Recent studies suggest a prevalence of SSc-PAH of at least 10% when the disease duration exceeds 10 years.^[15]^ Moreover, retrospective studies in monocentric cohorts have indicated an annual incidence of 1 to 2%, with a higher incidence of PAH in lcSSc patients and a slightly higher incidence of SSc-PH (postcapillary or ILD-associated) in dcSSc patients.^[16]^

## HRCT in ILD: Technical Consideration

**Figure 1:**
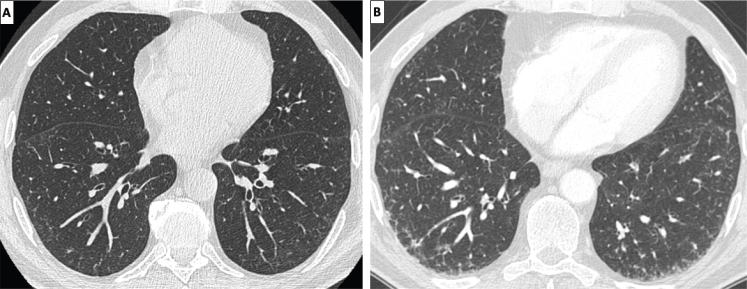
Example of chest HRCT images reconstructed prior (A) and after (B) contrast medium administration, demonstrating a generalized increase of lung density in B as compared to A. HRCT, high resolution computed tomography.

Appropriate acquisition and reconstruction of HRCT imaging is the first step to ensure a correct evaluation of patients with suspected ILD.^[17,18]^ Although there are different manners to perform HRCT imaging, key technical aspect is the use of thin slice thickness (0.5–1.5 mm) and high spatial frequency (or “sharp”) reconstruction algorithms. Inappropriate reconstruction algorithms and image display settings (window level of -600 to -700 Hounsfield Unit, HU, and window width of 1000 – 1600 HU are considered appropriate) as well as the use of contrast media contribute to misinterpretation of ILD-related abnormalities. Notably, inappropriate reconstruction algorithms might alter spatial resolution, while inadequate window level or width could create fictitious parenchymal abnormalities or mask pathological changes in lung attenuation.^[18]^ The use of iodinated contrast media causes a generalized increase of lung density that might influence the assessment of lung attenuation (Figure 1).^[19]^

The volumetric scanning of the whole chest with the patient lying in supine position at the end of a full inspiration should be followed by expiratory acquisition in case of inhomogeneous lung attenuation (*e.g*., mosaic attenuation pattern) to rule out air-trapping. Additional scanning with the patient lying in prone position should be also considered to differentiate between gravitational opacities and subpleural ground glass opacities (GGO) /fine reticulations, potential expression of interstitial lung abnormalities (ILA).^[20]^

Innovation technologies have progressively moved towards radiation dose reduction strategies. If in other fields of thoracic imaging, such as lung cancer screening, the use of low-dose and ultra-low dose techniques has shown excellent results, their application should be considered with caution in ILD. According to the latest recommendations, “low dose” protocols (*e.g*., 1–3 mSv) should be considered, whereas “ultra-low dose” protocols (< 1 mSv) ought to be avoided.^[21]^

## Parenchymal Involvement in Systemic Sclerosis

It is now commonly accepted that all incident SSc patients should undergo HRCT at baseline to screen for SSc-ILD. While in the past there was a huge heterogeneity of this crucial topic, two recent consensus papers have both agreed that HRCT should be performed in all new SSc patients.^[22,23]^ The reason beyond this statement is that ILD is generally an early complication of SSc, frequently occurring during the first 4-6 years after the onset of the disease.^[24]^ Nonetheless, a later onset has also been observed (*i.e*., at 8 years).^[25]^

**Figure 2:**
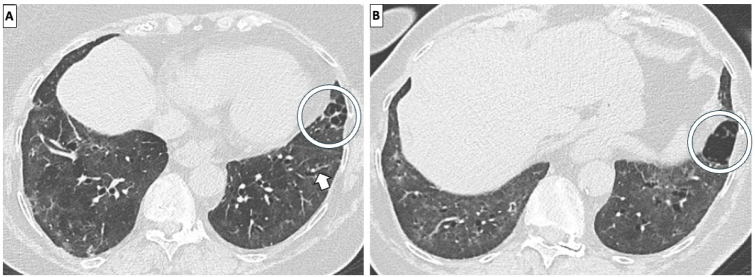
SSc-fibrotic NSIP demonstrated by diffuse bilateral ground glass opacities and some traction bronchiectasis (arrow). Parenchymal emphysema is also present (circles). SSc-fibrotic NSIP, systemic sclerosis-fibrotic nonspecific interstitial pneumonia.

Nonspecific Interstitial Pneumonia (NSIP) is the most common HRCT pattern observed in SSc patients, followed by the Usual Interstitial Pneumonia (UIP) pattern, as shown by the largest study of biopsy-proven SSc-ILD, where 77% of the patients had NSIP (mostly fibrotic) and the rest presented with UIP.^[26]^ Cellular NSIP, rarer in SSc, shows GGO with peribronchovascular distribution and lower lobe predominance, while fibrotic NSIP is demonstrated by bronchiectasis/bronchiolectasis, reticulation and architectural distortion with a lower lobe predominance, normally associated with a variable extent of GGO^[27]^ (Figure 2). Analogously to idiopathic NSIP and other connective tissue disease (CTD)-related NSIP, GGO corresponds to interstitial inflammation in the cellular form and to fibrosis in the fibrotic form. GGO may initially represent the predominant or the only abnormality. Limited honeycombing might also be present, seen more frequently in the limited form than in the diffuse cutaneous form of SSc.^[28]^

UIP pattern, the hallmark of idiopathic pulmonary fibrosis (IPF) is defined by the presence of honeycombing with a subpleural and lower lobe predominance, with or without peripheral bronchiectasis/bronchiolectasis and occasional GGO, while probable UIP pattern is demonstrated by the presence of subpleural, basal-predominant reticular abnormalities with peripheral traction bronchiectasis/bronchiolectasis; GGO can also be present. HRCT features of fibrosis (*i.e*., honeycombing, traction bronchiectasis/bronchiolectasis, reticulations and volume loss) that do not meet the criteria for UIP or probable UIP and do not suggest an alternative diagnosis should be regarded as “indeterminate UIP”,^[21]^ less commonly observed in SSc. As observed by Walsh *et al*.,^[29]^ the UIP pattern is associated with a worse prognosis in CTD, underlying the prognostic significance of the HRCT characterization.

**Figure 3:**
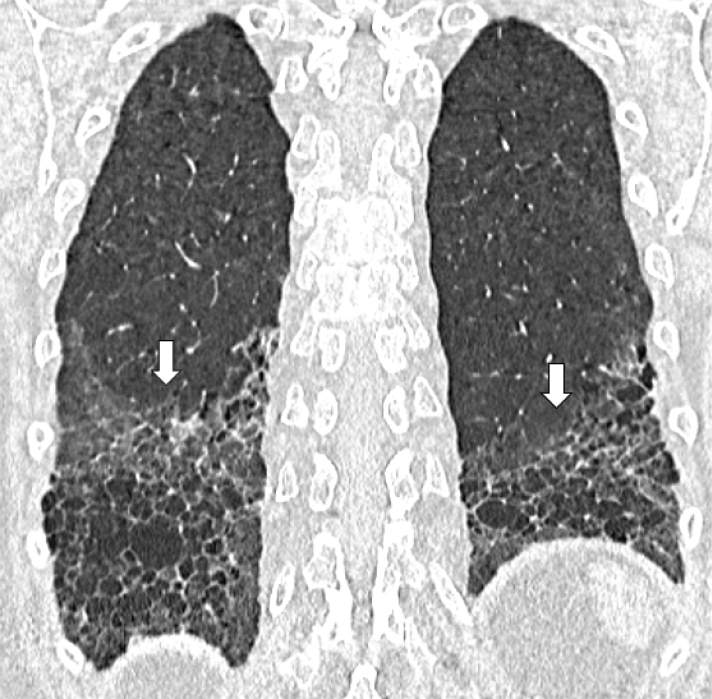
Coronal HRCT image of a case of SSc-UIP where fibrosis is predominantly located in the lung bases with a sharp and clear demarcation (arrows) in the cranio-caudal plane (“straightedge” sign). HRCT, high resolution computed tomography; SSc-UIP, systemic sclerosis-usual interstitial pneumonia.

Although idiopathic-NSIP and UIP are usually undistinguishable from secondary forms at HRCT imaging, some characteristic signs have been more recently described in CTD-related fibrotic ILDs, including SSc.^[27]^ These signs include the so-called “straight-edge” sign, consisting in isolated fibrosis predominantly located in the lung bases with a sharp and clear demarcation in the cranio-caudal plane^[30]^ (Figure 3), and “four corners” sign, defined by the presence of signs of fibrosis in the “4 corners regions of the thorax”, namely the anterolateral regions of mid-upper lobes and the posterosuperior regions of lower lobes.^[31]^ The former is thought to be related to the peribronchovascular distribution of fibrotic changes, mostly in NSIP cases, whereby as disease progresses, fibrosis extends out to the lateral margins of the lung, defining such a “straight-edge” sign.^[30]^ The pathophysiology of the “four corners” sign is still unknown; several underlying mechanisms have been suggested, including the distribution of lymphoid tissue in the lung, the presence of antibodies that would target these specific areas of the lungs and environmental factors.^[31]^

Other HRCT patterns described in SSc include diffuse alveolar damage (DAD), diffuse alveolar hemorrhage (DAH) and organizing pneumonia (OP), all represented by consolidation and/or GGO of variable distribution and extent.^[32]^

The extent of parenchymal involvement at HRCT correlates with patient prognosis. Goh *et al*. demonstrated that patients with a lung involvement < 20% had an average 10-year survival of 67%, whereas those with an involvement > 20% an average survival of 43%.^[33]^

## Extra-parenchymal Findings in Systemic Sclerosis

PH is a severe and relatively frequent complication of SSc, observed in 5 to 19% of cases.^[34]^ PAH is the most common cause of SSc-associated PH, with other causes including myocardial fibrosis, left heart disease, fibrotic ILD, and pulmonary veno-occlusive disease (PVOD).^[35]^

Indirect signs of PAH depictable on conventional CT imaging are mostly represented by the enlargement of the main and proximal pulmonary arteries. More specifically, dilatation of the main pulmonary artery (MPA) is a sensitive and specific indicator of PH and a MPA diameter > 2.9 cm has been shown to have an 89% specificity for diagnosing PH. Additionally, an MPA to ascending aorta diameter ratio over 1.0 serves as a sensitive predictor of PH. A dilated right or left pulmonary artery exceeding 1.8 cm also suggests PH and is a predictor of mortality.^[36]^

A nondilated pulmonary artery, however, does not necessarily exclude PAH. Other signs include cardiomegaly, pericardial thickening, and pericardial effusion, with pericardial involvement being considered a stronger predictor of echocardiographic PAH.^[37]^ CT signs of PVOD include mosaic attenuation, which reflects the patchy hyperemia and oligemia, smooth interlobular septa thickening, pleural effusion, and enlarged central pulmonary arteries.^[38]^

Newly developed quantitative imaging approaches have shown promising results in the non-invasive investigation of pulmonary vessels. Notably, some Authors have recently demonstrated a reduced vascular volume in lung regions with higher ILD extent in SSc patients.^[39]^

A detailed discussion of the numerous potential applications of artificial intelligence-based approaches in ILD, including SSc-ILD, goes beyond the aim of this review.

Esophageal involvement, mostly demonstrated by esophageal dilatation on CT imaging, is an early manifestation of SSc, found in up to 97% of patients. Esophageal dysmotility can lead to aspiration bronchiolitis or pneumonia, presenting with centrilobular nodules, consolidation, bronchiectasis, and/ or mucous plugging in the dependent portions of the lungs.^[40]^

Airway involvement without signs of fibrosis is rare in SSc when compared to other CTDs such as rheumatoid arthritis.^[28]^ Ntoumazios *et al*. reported a prevalence of 59% of cylindrical bronchiectasis in SSc patients screened with HRCT.^[41]^ The clinical significance of these results, however, is unclear. A pathogenetic link with recurrent aspiration pneumonia due to esophageal dysmotility can be suggested.

Multiple mediastinal lymphadenopathies are frequently observed in patients with SSc-ILD (in up to 60% of cases), and the degree of lymph node enlargement is thought to be related to the extent of the parenchymal involvement.^[42]^ Enlarged lymph node are usually not bulky nor compressive in SSc, and their number tends to remain stable over time. Renaud *et al*. have recently demonstrated that in the absence of ILD, thoracic lymphadenopathy seems to be more common in men SSc patients and in those with a history of exposure to silica.^[43]^

## Follow-up Evaluation

Chest HRCT has a well-established role in formulating an initial diagnosis of ILD. However, its ability to monitor patients with serial examinations can be considered equally important. As thoroughly discussed by Elicker *et al*., longitudinal HRCT evaluation provides relevant information on disease progression, occurrence of acute exacerbations and complications, and it also helps in defining the prognosis and in increasing the accuracy of initial diagnosis.^[44]^

Although the above considerations apply to SSc-ILD, no clear recommendations exist on whether and when a follow-up chest CT should be performed in SSc patients. Indeed, the occurrence of new or worsening respiratory symptoms with or without a pulmonary function decline is considered a valid indication for repeating chest CT, but whether a follow-up imaging assessment should be performed regardless of clinical and/or functional changes is still debated.^[22,23]^ Notably, there is evidence that 12–24-month follow-up chest HRCT might detect minimal but relevant changes in lung fibrosis extent in patients with stable pulmonary function.^[45]^

Moreover, HRCT can also play an important role in the follow-up of patients with PH as right heart remodeling secondary to chronic pulmonary pressure overload can be identified thanks to some specific HRCT features. More specifically, common indicators include right ventricular (RV) hypertrophy (wall thickness greater than 5 mm) and a dilated RV with an RV/Left ventricular ratio exceeding 1.0 in the axial plane. Additional findings may include a flattened interventricular septum, contrast reflux into the inferior vena cava (IVC), and IVC dilation.^[46]^

## Progression and Acute Exacerbation of ILD

Regardless of the underlying cause, fibrotic ILD are at a higher risk of progression and those presenting with a UIP pattern at baseline are at the highest risk.^[47]^ However, IPF-UIP has a worse prognosis than non-idiopathic UIP.^[44]^ Park *et al*. reported a 5-year mortality of 82% in IPF-UIP versus a rate of 45% in CTD-associated UIP.^[48]^

Progression of fibrosis is typically assessed visually, and radiological evidence of progression is demonstrated by at least one of the following: (i) increased extent or severity of traction bronchiectasis/bronchiolectasis, (ii) new GGO with traction bronchiectasis, (iii) new fine reticulation, (iv) increased extent or increased coarseness of reticular abnormality, (v) new or increased honeycombing and (vi) increased lobar volume loss.^[49]^ There is a large body of literature discussing the limitations of a mere visual assessment, which is well-known to be prone to high interobserver variability and affected by different techniques of imaging acquisition. New quantitative approaches have shown the potential to overcome some limitations,^[50]^ but their applicability remain limited in a clinical setting.

**Figure 4:**
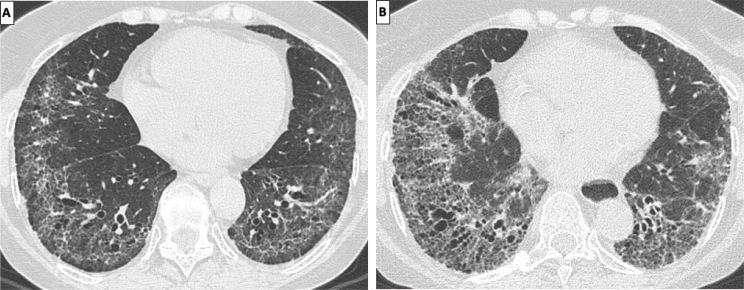
Axial HRCT images of a SSc-NSIP (baseline evaluation in A) showing evidence of fibrosis progression at 3-year follow-up (B), mostly demonstrated by an increased extent of traction bronchiectasis and reticular opacities. HRCT, high resolution computed tomography; SSc-fibrotic NSIP, systemic sclerosis-fibrotic nonspecific interstitial pneumonia

If cellular NSIP can resolve at follow-up HRCT, fibrotic NSIP is expected to worsen over time^[44]^ (Figure 4). Although progression of fibrotic NSIP is more commonly demonstrated by an increased extent and/or severity of traction bronchiectasis/ bronchiolectasis and reticulations, a minority of patients can present signs of evolution toward a UIP pattern over time.^[51]^

Progression of fibrosis in UIP pattern is normally demonstrated by an increased extent or severity of honeycombing, while in probable UIP new honeycombing can be observed.^[44]^

Acute exacerbation (AE) of ILD is a severe condition that frequently affects IPF-patients, accounting for 30%–40% of deaths. Albeit rarer, AE can occur in CTD, including SSc-patients, with similar prognostic impact.^[52]^ Sing *et al*. reported an AE-related mortality rate of 23% in a CTD-patients cohort, whereby most patients had diffuse cutaneous SSc. Increasing age and fibrotic ILD, mostly presenting with a UIP pattern, seem to be the most significant risk factors for the occurrence of AE.^[53]^ The CT manifestations of AE include bilateral ground-glass opacity and/or consolidation superimposed on a background pattern of fibrotic ILD.^[54]^

## Complications and Association with Other Conditions

Some studies reported an increased risk for lung cancer, mostly adenocarcinoma, in SSc patients, including nonsmokers,^[55]^ whereas others have not shown an increased risk.^[56,57]^ The occurrence of lung cancer seems to be more frequent in SSc patients with fibrotic ILD, suggesting that the risk factor for lung cancer is represented by the lung fibrosis rather than by the disease itself.^[58]^

Rare associations with other entities, such as sarcoidosis and pleuroparenchymal fibroelastosis (PPFE) have been described. Sarcoidosis in SSc is rare but appears to occur more frequently than in the general population. Himmel *et al*. reported that sarcoidosis tends to precede SSc manifestations and seems to be associated with pulmonary, lymph node, cutaneous, joint, and hepatic involvement.^[59]^

PPFE is a clinical-pathological entity of unknown etiology, affecting the visceral pleura and the subpleural parenchyma with an upper-lobe predilection.^[60]^ Bonifazi *et al*. have recently observed a prevalence of 18% of PPFE in a large cohort of SSc patients, demonstrating that the presence of PPFE was independently associated with a mortality risk of 77% on a multivariable analysis including, among other factors, age, Goh *et al* staging system, progressive functional decline, and PH.^[60]^ Similarly, a Japanese study showed an overall prevalence of PPFE in CTDs of 19%, with the highest peak in the SSc subgroup (43%). Authors have also observed that PPFE was a significant risk factor for mortality secondary to respiratory causes.^[61]^

## Conclusions

ILD and PH are the leading causes of death in SSc patients. Due to the non-specific or even asymptomatic nature of lung involvement at the early stages of disease, pulmonary involvement can go undiagnosed. Clinicians need to have a low threshold for ILD and PH evaluation in SSc patients and both clinicians and radiologists should be aware of the role of CT imaging, which is of utmost importance at both the diagnosis and follow-up of SSc patients with lung involvement.
